# Closure of Forensic Psychiatric Hospitals in Brazil and Its Impact on the Training of Future Psychiatrists in the State of Alagoas: An Experience Report

**DOI:** 10.1192/j.eurpsy.2026.11973

**Published:** 2026-07-31

**Authors:** L. C. Carneiro, M. I. O. de Morais, A. F. C. Ximenes, L. V. de Araújo, P. F. B. de Araújo, A. B. D. C. Alves, F. O. Barbosa, B. D. A. A. L. T. D. Melo, V. L. D. Melo-Neto, D. B. B. Ferreira, E. D. F. Correia

**Affiliations:** 1 Universidade de Ciências da Saúde de Alagoas; 2 Universidade Federal de Alagoas, Maceió, Brazil

## Abstract

**Introduction:**

Forensic psychiatric hospitals are specialized institutions designated for the custody and treatment of individuals diagnosed with mental disorders who have committed criminal offenses and are deemed a risk to public safety. This often reinforces stigma, weakens therapeutic bonds, and complicates reintegration. In 2024, psychiatry residents in Alagoas, Brazil, trained in such a facility and later participated in the reintegration process following its closure, in line with national anti-asylum policies promoting community-based care.

**Objectives:**

To describe the experience of psychiatry residents during and after the closure of a forensic psychiatric hospital, emphasizing the role of humanized, multidisciplinary care and the importance of understanding legal, clinical, and social aspects involved in the treatment of individuals in conflict with the law.

**Methods:**

Residents received supervised training at the forensic hospital alongside an interdisciplinary team. After the hospital’s closure, they joined the Evaluation and Monitoring Team for Therapeutic Measures Applied to People with Mental Disorders in Conflict with the Law (EAP), supporting the transition of patients to community-based services such as Psychosocial Care Centers (CAPS).

**Results:**

The hospital’s closure marked a shift from institutional to community care. Residents observed challenges related to stigma, treatment adherence, and the rebuilding of therapeutic alliances. Patients, often institutionalized for years, faced difficulties reintegrating into society. The experience highlighted the importance of continuous, interdisciplinary care and the psychiatrist’s role in promoting recovery, autonomy, and social inclusion.

**Conclusions:**

This experience was transformative for psychiatric training. It deepened residents’ understanding of the intersection between mental health and the justice system and reinforced the value of a humanized, community-based approach. The transition from custodial care to reintegration underscored the need for long-term therapeutic support and respect for patients’ rights, aligning clinical practice with ethical and legal principles in forensic psychiatry.

**Disclosure of Interest:**

None Declared

